# Block-based teaching method based on cybernetics: a trial with 115 Chinese undergraduate medical students

**DOI:** 10.1186/s12909-023-04643-0

**Published:** 2023-09-11

**Authors:** Zhi-Ping Liu, Si-Han Liu, Xin Dai, Jie Chen, Qing-Feng Guo, Da-Xin Zhang

**Affiliations:** 1 Department of Oncology, The First Affiliated Hospital of Harbin Medical University, 23 Youzheng Street, Nangang District, Harbin, 150001 Heilongjiang Province China; 2 Department of Academic Affairs, The First Affiliated Hospital of Harbin Medical University, 23 Youzheng Street, Nangang District, Harbin, 150001 Heilongjiang Province China

**Keywords:** Cybernetics, Block-based teaching method, Undergraduate, Medical student, China

## Abstract

**Background:**

Class attendance is important for academic performance. Personal interactions between teachers and students are difficult in large classes; the number of medical undergraduate students in China ranges from dozens to over 100. It is important for teachers to control the teaching process to improve student attendance and participation.

**Methods:**

Two classes of fourth-year undergraduate medical students, with each class comprising 115 students, participated in the study. One class, the trial group, was taught by the block-based teaching method based on cybernetics. This study was conducted with three of the courses in the Introduction to Oncology subject, and the trial group’s courses included several blocks. Each block had a test paper that the students responded to immediately in class using the Internet. The teacher obtained feedback from the students when the rate of correct responses to block-test questions was less than 90%. The teacher adjusted the teaching in the following blocks according to the feedback information. The other class, the control group, was taught using the traditional lecture-based teaching method.

**Results:**

The average attendance in the trial group was 104/115 (90.43%), and that in the control group was 83/115 (72.17%) (*p* = 0.0003). The teacher adjusted the teaching three times in the radiotherapy course owing to the complex ideas. After feedback, information on chemotherapy for the upper body was adjusted once, as was that on chemotherapy for the lower body, owing to students’ attitudes. The average total score of the trial group was 86.06 ± 17.46 and that of the control group was 80.38 ± 6.97 (*p* = 0.041). Questionnaire I showed that the trial group students’ attendance and participation were better than in the control group. Questionnaire II showed that the block-based teaching method based on cybernetics was approved by the students.

**Conclusions:**

The block-based teaching method based on cybernetics used in medical classes with large numbers of Chinese undergraduate students had positive effects.

## Background

Class attendance is important for undergraduate medical students, because it is positively correlated with academic performance [1, 2]. Research has shown that the reasons for medical students’ absences included skipping a less important course to study for a larger-credit course and preparing for more important exams [2]. Furthermore, active class participation, defined as students’ comments or questions in class related to the course content, contributes to student learning [3]. Students are encouraged to actively express their ideas [3], but it is impossible for a teacher to communicate with every student in a large class of 100 or more students. The Introduction to Oncology subject is a smaller-credit course in the target medical university, and it faces serious absenteeism issues. The medical undergraduate classes in this university have over 100 students, and students rarely participate in class. In order to improve the quality of teaching of Introduction to Oncology, it is extremely important for the teacher to control the teaching process to improve the attendance and participation of the class.

Chinese scholars have applied the block-based teaching method to control the teaching process [4, 5]. This method divides the class content into multiple blocks, and each block is composed of one topic. In each block, teaching, interaction between teacher and students, and block tests are set up. Multiple block tests prompt every student to answer, allowing the teacher to control the entire class process [4, 5]. Zhang et al. applied the block-based teaching method to teach Soil Mechanics to undergraduates and postgraduates in Tsinghua University to address the lack of self-discipline of students in an online, unruly environment [4]. Likewise, influenced by the COVID-19 pandemic, Li et al. used the block-based teaching method for the online teaching of a Power Engineering Testing Technology course. Students’ classroom participation, attendance, and learning enthusiasm improved [5].

Block-based teaching involves the simple control of the teaching process without adjusting the process itself. It is necessary to integrate cybernetics into block-based teaching so that teachers can obtain feedback information from students’ performance and adjust their teaching accordingly. The term “cybernetics” was first proposed in 1948 by American mathematician Norbert Wiener [6], who defined cybernetics as the art of governing. This term is derived from the Greek word “kubernetes,” which means steersman. To reach a destination, the steersman uses tools to change the boat’s direction. The steersman’s choice of tools depends on environmental variables such as the wind and waves, which keep the boat from reaching the target. During the entire process, the steersman adjusts the ship’s direction according to changes in external conditions to arrive at the destination. Cybernetics investigates regulatory systems including their structures, constraints, and possibilities. It has been widely used in machinery, physics, biology, cognition, social systems, and other fields [6]. In 1973, Ted Cantrell proposed the cybernetics model of teaching [7]. He believed that in a one-to-one situation between teacher and student, the teacher should constantly adjust the speed of speech and language style based on the student’s feedback signals (expressions, body language, answers to questions) to make the student more interested and enthusiastic. When teachers face multiple students, the students’ reactions (feedback signals) are all different. In such a situation, the group of students needs to be considered as a whole when obtaining feedback and making teaching adjustments.

In order to make full use of classroom time to improve students’ learning efficiency in Introduction to Oncology, we applied the block-based teaching method based on cybernetics to manage the large numbers of Chinese medical students and observe the students’ class attendance and participation.

## Method

### Study setting

Introduction to Oncology is taught in the 4^th^-year curriculum of the First Affiliated Hospital of Harbin Medical University. The subject includes etiology, diagnosis and comprehensive treatment, radiotherapy, chemotherapy (upper body and lower body), biological therapy, traditional Chinese medical treatment, and palliative and analgesic treatment. There are eight courses in total, each lasting 90 min. All courses until now have been taught using a lecture-based teaching method. At the time of this research, there were 230 4^th^-year undergraduate medical students; they were divided into two classes (115 students each) because of the large size of the cohort. Both classes took the same courses with the same teachers in their regular curriculum.

### Study design

**Fig. 1:**
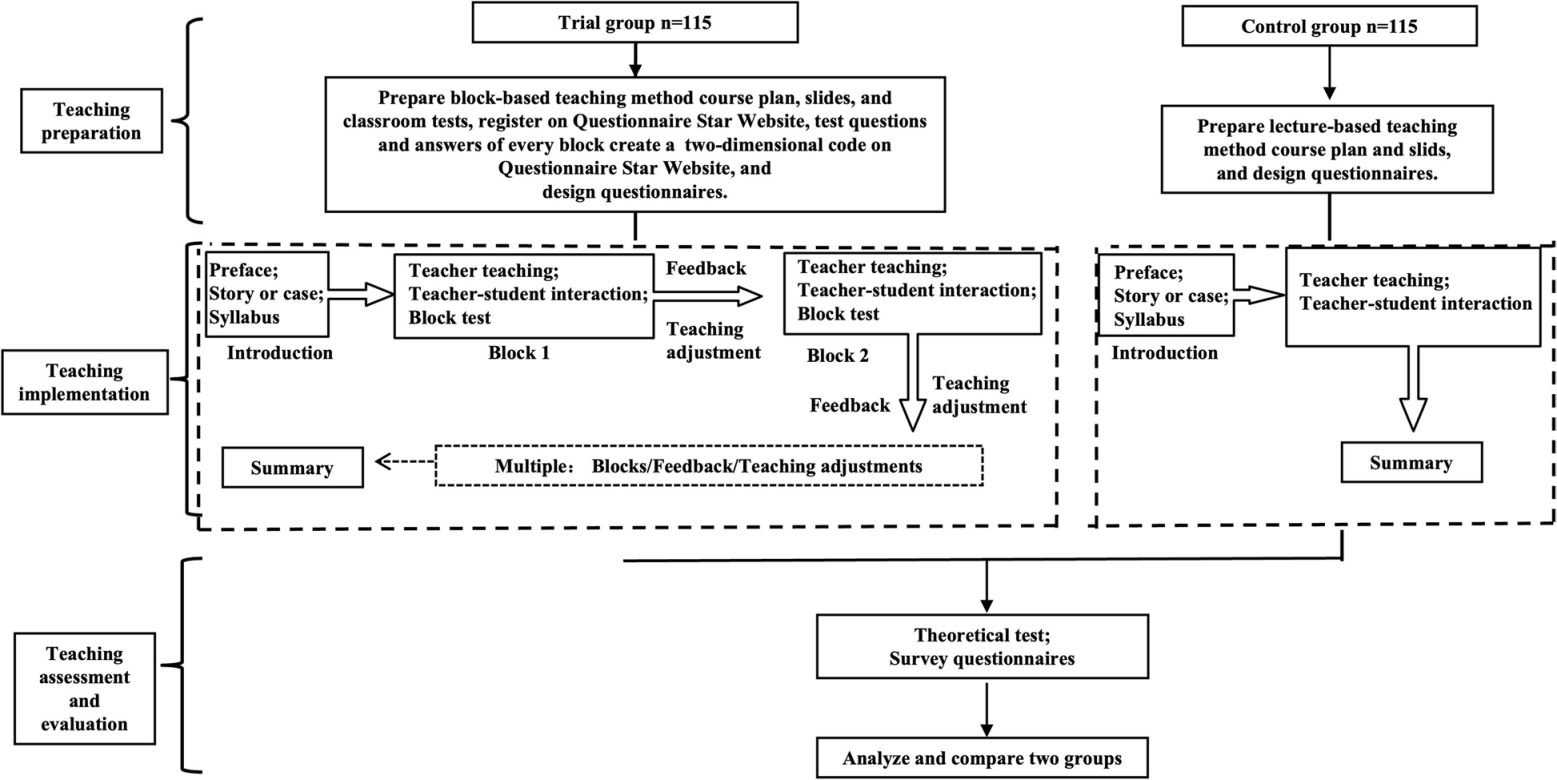
Teaching flow chart

During the second half of 2022, all 4^th^-year undergraduate medical students participated in this pilot study. One class was used as the trial group and the other class as the control group. There were no statistical differences in their age, gender, scores on the entrance exams, or school performance between the two groups. The trial group was taught using the block-based teaching method based on cybernetics, while the control group was taught using the lecture-based teaching method. This study was conducted in three courses, including radiotherapy, chemotherapy for upper body, and chemotherapy for lower body. The three courses for both groups were taught by the same teacher. The flows of the trial group and control group are shown in Fig. 1. The two group students’ other five courses were taught by the lecture-based teaching method as usual. This study was reviewed and approved by the Clinical Research and Research Ethics Committee of the First Affiliated Hospital of Harbin Medical University (ethical review: 202381). All students provided informed consent before enrolling in this study. All the procedures performed in this study were in accordance with the principles of the Declaration of Helsinki.

#### Teaching preparation

For the trial group, the teacher prepared course slides, lesson plans, and classroom test questions and answers based on the block-based teaching method. The teacher registered an account on the Questionnaire Star website (https://www.wjx.cn). Every course had three or four block tests. Each test generated a two-dimensional code on the Questionnaire Star website, and each two-dimensional code was put into each block of the course slides. For the control group, the teacher prepared course slides and a lesson plan using the lecture-based teaching method. The teacher designed Questionnaire I pertaining to the students’ attendance and participation in class and Questionnaire II pertaining to the students’ evaluation of the teaching mode.

#### Teaching implementation

For the trial group, each course included three parts: introduction, main body, and summary. The teacher introduced herself (preface), presented a story or a clinical case to introduce the course, and shared the syllabus in the introductory section. The main body was divided into three to four blocks. Each block had a theme of approximately 10–20 min (depending on the content of the block). Each block included teaching, teacher–student interactions, and students answering block test papers (with one to five questions). The teacher presented the two-dimensional code of the block test papers when students were required to respond to the block test. The students scanned the two-dimensional codes with their mobile phones on the WeChat app, answered the questions, and submitted test papers to the Questionnaire Star website. Each test paper was completed within 2–3 min. The teacher instantly obtained the scores of every student and the rate of correct answers to every question on the Questionnaire Star website. When the rate of correct answers to one or more questions in the block test was less than 90%, the teacher obtained feedback (typically through communicating with students) and adjusted their teaching in the following blocks. The teacher communicated with the students (online and on-site students) who responded with incorrect answers to understand the reasons they chose the incorrect answers. The teacher further obtained feedback by observing students’ expressions and body language (on-site students). Before the next block, the teacher summarized the problems encountered in feedback of the previous block and improved on them in the following blocks. That is to say, between the two blocks, the teacher received and summarized feedback and adjusted the teaching once. In the summary section, the teacher summarized the key points of the course and provided homework.

The control group also had three sections. The contents of the introduction and summary sections were similar to that of the trial group. The main section included teaching and teacher–student interactions. These interactions in both groups involved the teacher asking students questions randomly and the students answering the questions.

#### Teaching assessment

Once all the Introduction to Oncology courses had been completed, the final theoretical examination was held. Two questionnaire surveys were conducted pertaining to attendance and participation in class and evaluations of the teaching methods. The Introduction to Oncology subject had eight courses, and this trial was conducted in three of them. For the trial group, students had five courses taught by the lecture-based teaching method and three courses taught by the block-based teaching method based on cybernetics. For the control group, students had all eight courses taught by the lecture-based teaching method. Therefore, Questionnaire II, pertaining to students’ evaluation of the two teaching modes, was administered to the trial group but not the control group. Questionnaire I, pertaining to the students’ attendance and participation in class, was administered in both groups.

### Outcomes and evaluation

We summarized and compared the attendance numbers and overall scores of the two groups. The final exam scores of the radiotherapy and chemotherapy courses (three courses) were part of Introduction to Oncology and were converted into a 100-point system. The overall score of the control group was the final exam score of these three courses. For the trial group, the class tests accounted for 60%, and the final exam scores of these three courses accounted for 40%, of their overall score. The questions in these block tests were not related to questions on the final examination. The class tests and the final exams were designed by different teachers and contained different questions. This method of calculating scores was part of the block-based teaching method based on cybernetics. It was a coercive measure to induce students to attend and pay more attention to the class. The data for classroom attendance and participation in the two groups were obtained and compared through Questionnaire I. The students’ evaluations of the two teaching methods were obtained and compared through Questionnaire II.

### Statistical analysis

Normally distributed data were reported as means and standard deviations. The total scores of the two groups were compared using a single sample t-test. The chi-square test was used to compare class attendance numbers and responses of Questionnaire II pertaining to the students’ evaluation of the teaching mode between the two groups. The comparison of the responses in Questionnaire I pertaining to class attendance and participation between the two groups was based on the Kruskal–Wallis ANOVA test. All statistical analyses were conducted using SPSS Statistics (v.25; IBM Corporation, NY, USA). A two-tailed p value < 0.05 was considered statistically significant.

## Results

Given the COVID-19 pandemic, some students who participated were in the classroom; those at home used the WeChat Enterprise Edition, which was broadcast live. Students who were online could hear the voices of teachers and simultaneously saw slides. At the beginning of the three courses, the trial group was informed that class test scores would be included in the overall scores. The total number of attendees included the number of students attending the classroom and those online using the WeChat Enterprise Edition. The average attendance rate of the trial group was 104/115 (90.43%) and that of the control group was 83/115 (72.17%). There was a significant difference between the attendance rates of the two groups (*p* = 0.0003).

The results of the block tests of the trial group are summarized in Table 1. After the students answered the block test paper, the teacher received the block test score from Questionnaire Star website. If the rate of correct answers to one or more questions was less than 90%, the teacher immediately communicated with the students who had responded with incorrect answers. Furthermore, the teacher summarized students’ expressions and body language. The teacher adjusted the teaching three times in the radiotherapy course. First, the rate of correct answers to Questions 2 and 3 in Block I was less than 90%, primarily because understanding the characteristics of stereotactic radiation therapy and brachytherapy was challenging. The teacher immediately explained the two radiotherapy technologies in detail in the class and showed a short introductory video. The teacher added pictures or short videos in the following blocks to ensure that the knowledge could be easily grasped. Second, the rate of correct answers to Question 7 in Block II was 85.44%. This question was not difficult, and the poor results were mainly because the students did not pay attention in class. In the later blocks, the teacher increased random oral questions to increase interaction with the students and the use of examples or cases to induce students to participate in the class. Third, the rate of correct answers to Question 9 and 10 about radiotherapy dosages and indications for palliative radiotherapy in Block IV were less than 90% because the students did not understand these topics well. The teacher gave supplementary explanations on the differences of different radiation dose segmentation modes and the indications for palliative radiotherapy. In summary, the feedback and adjustment of the radiotherapy course indicated that the incorrect answers were mainly because of the difficulty of the topics.

**Table 1:** Block test results of the trial group

	Radiotherapy		Chemotherapy for upper body		Chemotherapy for lower body	
	Question number	Percentage of correct answers	Question number	Percentage of correct answers	Question number	Percentage of correct answers
Block I	1	92.16%	1	93.81%	1	91.75%
	2	89.22%			2	91.75%
	3	89.22%			3	91.75%
	4	97.06%			4	97.94%
					5	98.97%
Block II	5	94.17%	2	97.12%	6	92.93%
	6	97.09%	3	96.15%	7	85.23%
	7	85.44%	4	84.62%		
			5	97.12%		
			6	96.15%		
Block III	8	98.96%	7	99.03%	8	91.92%
			8	95.15%	9	98.99%
					10	90.91%
Block IV	9	85.63%	9	96.81%		
	10	88.16%	10	95.74%		

Feedback and adjustment were conducted once in chemotherapy for upper body owing to the 84.62% correct answer rate to Question 4 in block II. The reason for this result was that the teacher spoke too fast and knowledge points were dense according to students’ oral feedback and the teacher’s observation of students’ expressions and body language. The teacher made adjustments, speaking slowly and leaving time for students to understand in the subsequent blocks. Teaching was adjusted once during the chemotherapy for lower body topic because of Question 7 in Block II. The reason for the poor response was that some students did not pay attention in the classroom according to students’ oral feedback and the teacher’s observation of students’ expressions and body language. The teacher used random oral questions to interaction with the students to remind students to pay attention in class and included stories or case studies to make the classes more interesting. In summary, the feedback and adjustments made in the chemotherapy courses were designed to address the students’ attitudes in the class.

Comparing the overall scores of the two groups, the average score was 86.06 ± 17.46 in the trial group and 80.38 ± 6.97 in the control group (*p* = 0.041).

Questionnaire I, pertaining to the students’ attendance and participation in class, was distributed among the students of the two groups, with 230 copies being released and 230 copies being recovered (Table 2). There was no significant difference between the two groups regarding the answers to Questions 1–3. Most students thought that medical courses and exams were difficult and that the Introduction to Oncology course was necessary and helpful. Question 5 showed that no student was absent two or three times from all three courses in the trial group, in contrast to the 6.96% absence in the control group (*p* = 0.041). Question 6 required the absent students to explain reasons for their absence from the three courses. We did not compare the two groups’ data because they were descriptive multiple-choice answers. Questions 7–10 showed that in the trial group, more students paid attention to class (*p* = 0.007) and that fewer students were distracted by exam preparation for subjects other than Introduction to Oncology (*p* = 0.001), were engaged in entertainment with electronic products (*p* = 0.003), or faced absent-mindedness (*p* = 0.007).

**Table 2:** Questionnaire I pertaining to students’ attendance and participation in class

Questions	Groups	Options					*P*
		Strongly agree	Agree	Moderate	Disagree	Very disagree	
Q1. Do you think medical courses and exams are intensive, hard to cope with, and hard to learn?	Trial group (n, %^b^)	48, 41.74%	37, 32.17%	25, 21.74%	2, 1.74%	3, 2.61%	0.820
	Control group (n, %^b^)	40, 34.78%	57, 49.57%	18, 15.65%	0	0	
Q2. Do you think it is necessary to learn the course, Introduction to Oncology?		Very necessary	Necessary	Moderate	Unnecessary	Very unnecessary	
	Trial group (n, %^b^)	28, 24.34%	52, 45.22%	32, 27.83%	2, 1.74%	1, 0.87%	0.509
	Control group (n, %^b^)	20, 17.39%	76, 66.09%	19, 16.52%	0	0	
Q3. Do you think the course, Introduction to Oncology, is helpful to your medical profession?		Very helpful	Helpful	Moderate	Unhelpful	Very unhelpful	
	Trial group (n, %^b^)	31, 26.96%	62, 53.91%	19, 16.52%	0	3, 2.61%	0.702
	Control group (n, %^b^)	24, 20.87%	72, 62.61%	19, 16.52%	0	0	
Q4. How many times have you been late for all three courses of Introduction to Oncology?		None	Once	Twice	Three times		
	Trial group (n, %^a^)	336, 97.39%	3, 0.87%	6, 1.74%	0		0.300
	Control group (n, %^a^)	300, 94.78%	6, 1.74%	30, 0.87%	9, 2.61%		
Q5. How many times have you been absent from all three courses of Introduction to Oncology?		None	Once	Twice	Three times		
	Trial group (n, %^a^)	312, 90.44%	33, 9.56%	0	0		0.041
	Control group (n, %^a^)	282, 81.74%	39, 11.30%	15, 4.35%	9, 2.61%		
Q6. The reason why you are absent from the three courses of Introduction to Oncology (the absent student answers this question) (multiple choices)^c^		Preparing for exams in other subjects	Could cram for Oncology exam and pass it	Medical courses are tough, no time to attend every course	Absent due to other reasons	Class content can be boring, or teaching can be boring	
	Trial group (%)	42.10%	26.31%	26.31%	21.05%	5.2%	
	Control group (%)	36.36%	40.91%	54.54%	22.73%	9.09%	
Q7. What proportion of time did you pay attention to the three courses of Introduction to Oncology?		0–20%	20–40%	40–60%	60–80%	80–100%	
	Trial group (n, %^b^)	13, 11.30%	21, 18.26%	37, 32.17%	27, 23.48%	17, 14.78%	0.007
	Control group (n, %^b^)	26, 22.61%	21, 18.26%	38, 33.04%	26, 22.61%	4, 3.48%	
Q8. What percentage of time did you spend learning other subjects in the three courses of Introduction to Oncology?		0–20%	20–40%	40–60%	60–80%	80–100%	
	Trial group (n, %^b^)	58, 50.43%	30, 26.09%	21, 18.26%	6, 5.22%	0	0.001
	Control group (n, %^b^)	38, 33.04%	29, 25.22%	33, 28.69%	9, 7.83%	6, 5.22%	
Q9. What percentage of your time was spent checking mobile phones, playing mobile games, and online chatting in the three courses of Introduction to Oncology?		0–20%	20–40%	40–60%	60–80%	80–100%	
	Trial group (n, %^b^)	86, 74.78%	21, 18.26%	8, 6.96%	0	0	0.003
	Control group (n, %^b^)	65, 56.52%	33, 28.69%	11, 9.57%	6, 5.22%	0	
Q10. What percentage of your time was spent absent-minded in the three courses of Introduction to Oncology?		0–20%	20–40%	40–60%	60–80%	80–100%	
	Trial group (n, %^b^)	97, 84.35%	13, 11.30%	5, 4.35%	0	0	0.007
	Control group (n, %^b^)	80, 69.56%	24, 20.87%	7, 6.09%	4, 3.48%	0	

^a^Number of % is n ÷ 345

^b^Number of % is n ÷ 115

^c^The data of the two groups were not compared

Questionnaire II, pertaining to students’ evaluation of the teaching mode, was distributed among the students in the trial group. A total of 115 questionnaires were distributed, and 110 were recovered (Table 3). Most students responded that the block-based teaching method forced them to participate in the class (*p* < 0.001), forced them not to do other things in the class (*p* < 0.001), and enabled students to be noticed by the teacher (*p* < 0.001). Most students responded that the block-based teaching method made the teaching process interesting (*p* = 0.030), activated the classroom atmosphere (*p* = 0.003), made it easier to understand and remember the classroom content (*p* < 0.001), and improved their test scores (*p* < 0.001). Questions 1 and 5 showed that the two teaching methods had no difference in terms of being liked by students or in reducing fatigue (*p* > 0.05).

**Table 3:** Questionnaire II pertaining to students’ evaluation of teaching mode

Questions	Block-based teaching method (n, %)	Lecture-based teaching method (n, %)	*p*
Q1. Which teaching mode do you like?	55, 50.00%	55, 50.00%	1.00
Q2. Which teaching mode forced you to participate in the class?	90, 81.82%	20, 18.18%	< 0.001
Q3. Which teaching mode forced you to pay more attention to the class so that you were unable to do other things in the class?	88, 80.00%	22, 20.00%	< 0.001
Q4. Which teaching mode enabled you to be noticed by teachers?	85, 77.27%	25, 22.73%	< 0.001
Q5. Which teaching mode reduced your fatigue in the classroom?	59, 53.63%	51, 46.36%	0.280
Q6. Which teaching mode made the teaching process more interesting?	63, 57.27%	47, 42.73%	0.030
Q7. Which teaching mode activated the classroom atmosphere?	66, 60.00%	44, 40.00%	0.003
Q8. Which teaching mode made it easier for you to understand and remember the classroom content?	78, 70.91%	32, 29.09%	< 0.001
Q9. Which teaching mode improved your test scores?	79, 71.82%	31, 28.18%	< 0.001

## Discussion

We combined cybernetics using the block-based teaching method. The results of this study showed that the block-based teaching method based on cybernetics enabled the teacher to control the class at a high level throughout the teaching process and improved students’ class attendance and participation. This teaching method was approved of by the students.

This study showed that medical undergraduate students in China experience extreme academic pressure. Findings from Questionnaire I of this study reveal that more than 70% of students in both groups thought that the medical courses and exams were intensive, hard to cope with, and covered topics that were hard to learn. The control group learned from the traditional lecture-based teaching method, and its data reflected the routine status. The attendance rate was only 72.17%. The main reason for class absence was that 54.54% of students had no time to attend every course because of other medical courses with heavy workloads, 40.91% of students crammed for oncology in order to pass, and 36.36% of students were preparing for other subjects’ exams. From Question 8 of Questionnaire I, we found that 28.69% of students who came to the Introduction to Oncology class spent 40%–60% of their time studying other subjects. With 115 students in the Introduction to Oncology class, and facing issues of absenteeism and low participation, it is extremely important for teachers to encourage students to join the classroom and improve the quality of teaching, maximizing students’ knowledge during the class time.

The goal of education is to improve students’ knowledge and their ability to acquire knowledge. Attendance and participation in class are essential indicators for elevating students’ academic performance [8]. Scholars have adopted various methods to improve class attendance and participation. Wright et al. gave students participation paper tickets and entered students into a drawing for gift certificates. In this way, attendance and participation were improved for a second-year psychology class of undergraduates [3]. Purnama et al. used memes and Instagram to promote students’ engagement in classroom activity according to characteristics of the youth who tended to be visual and attracted to rapid information [9]. In this study, class test scores were included in the overall Introduction to Oncology score to induce students to attend classes, and class tests were used to encourage students to focus and leave them no time to engage in activities irrelevant to the class in the trial group. The attendance rate of the trial group was 90.43%, which was significantly higher than that of the control group (72.17%). Furthermore, Questionnaire I showed that in the control group, 6.96% of students were absent two or three times from all three courses, whereas no students were absent from the trial group (*p* = 0.041). Questions 7–10 of Questionnaire I showed that the trial group had more participation in class. The overall scores of the trial group were higher than those of the control group. Therefore, the block-based teaching method based on cybernetics is shown to improve students’ attendance and participation and increase students’ examination scores.

**Fig. 2:**
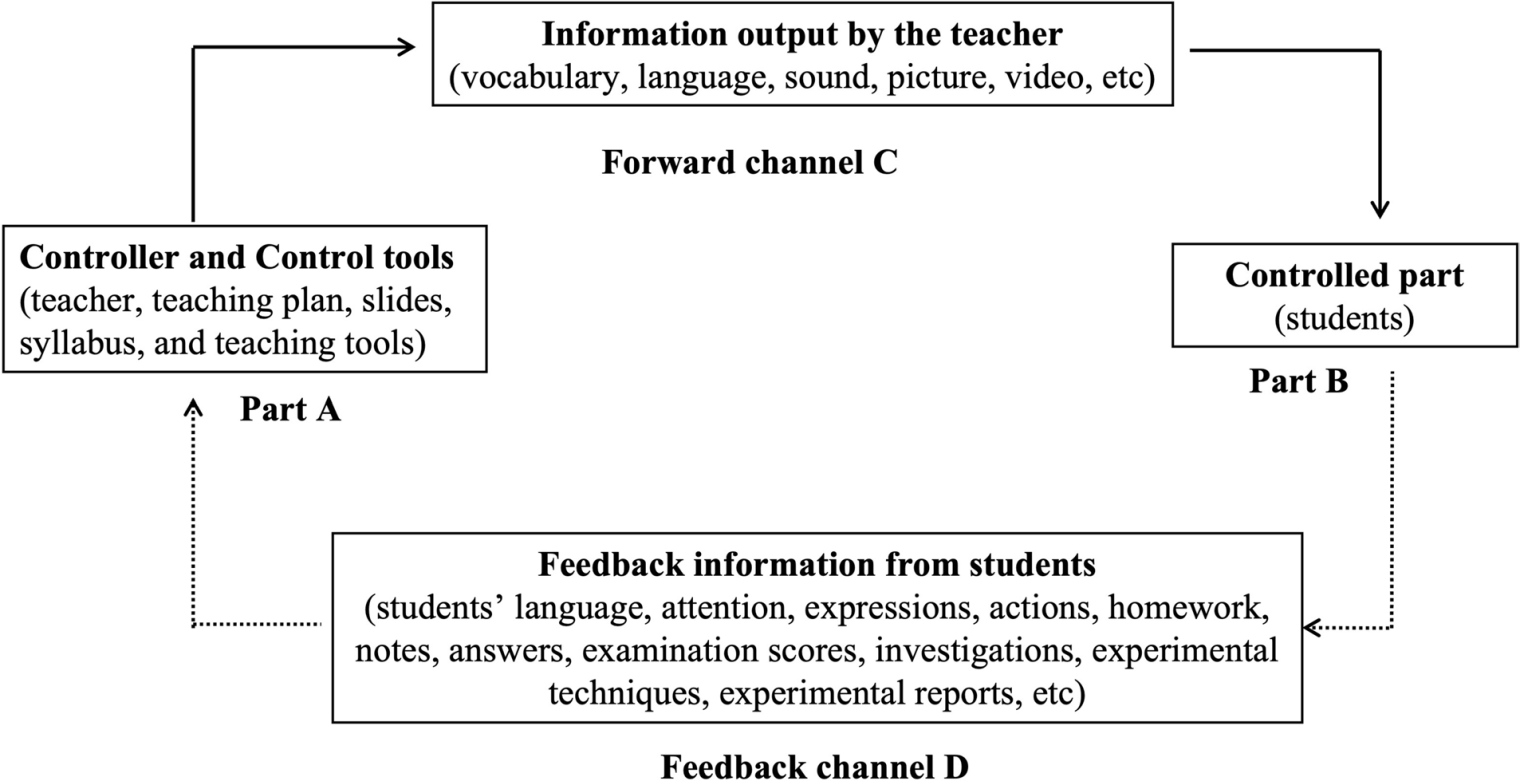
Simple system model diagram of teaching cybernetics

Teaching cybernetics in this study can be summarized as a simple system model as in Fig. 2 [10]. Part A is the controller and control tools. The controller is the specific executor of the teaching activities, the teacher. The control tools, the basis for controlling activities, are the teaching plan, slides, syllabus, and teaching tools. The forward channel C (solid line) is vocabulary, language, sound, picture, and video information output by the teacher. The controlled part B is the student who receives the information from the control part (Part A). Feedback channel D (dotted lines) refers to all feedback information from students. The feedback information includes the students’ language, attention, expressions, actions, homework, notes, answers, examination scores, investigations, experimental techniques, and experimental reports. The teacher adjusts the teaching activities according to the feedback information and transfers the teaching activities to the students through the forward channel C [10]. This cycle is used to achieve the predetermined teaching objectives (the balanced state of the system). The difficulty of the teaching cybernetics model is on how to obtain feedback, especially for classes of approximately 100 students. In this study, we took the question scores as an indicator to obtain feedback. If the correct rate for a question was lower than 90% for the entire group, we obtained oral feedback from the students and noted their body language and adjusted the subsequent teaching. Therefore, we maintained the teaching process at a high level, with more than 90% of students mastering knowledge points.

Since its inception, the model of teaching cybernetics has gradually been applied to college teaching, and several reports on the use of cybernetics in teaching methods have been written. For example, Peña-Ayala et al. used a ubiquitous learning strategy based on cybernetics having two characteristics: goal-directed and self-regulating. Using this strategy, students developed, applied, and self-regulated their learning actions to acquire domain-knowledge and develop their learning skills [11]. Terekhov created an education project at St. Petersburg State University in Russia called the cybernetics construction set, which was used for robot programming training for college students and achieved success [12]. Zhang applied cybernetics in the teaching method of physiology, set the teaching process as a cybernetics system, and adjusted the details of teaching according to feedback information from students. This improved the teaching quality [10].

Previous studies have shown that most feedback in the teaching cybernetics system is delayed (e.g., questionnaires or exams). Previous cybernetics systems took a long time to be completed; feedback and correction took place only once [13]. In this study, we divided the teaching content into several blocks, with each block being a cybernetic system. The block tests were designed for instant feedback using Internet technology. Instant feedback ensured that multiple cybernetics systems could be run in a class and that multiple instances of feedback and teaching adjustments ensured the high quality of the teaching process. In this study, attendance rates, the overall scores of the three courses, and teaching model evaluation questionnaires confirmed that the block-based teaching method based on cybernetics improved students’ class attendance and participation and was approved of by students.

This study has some limitations. First, the sample size of this study was small. Second, it was a single-center study. Third, part of the feedback information was obtained from oral communication with students. It was difficult for students to give the teacher their reason for getting a wrong answer when they did not pay attention in class. In future studies, we will focus on improving the methods for obtaining feedback from large classes to develop a more mature teaching cybernetics system.

In conclusion, the block-based teaching method based on cybernetics had positive effects when used in medical classes with large numbers of Chinese undergraduate students.

## Data Availability

The datasets generated and/or analyzed during the current study are available from the corresponding author on reasonable request.

## References

[1] Allen CW, Diamond-Myrsten S, Rollins LK (2018). School absenteeism in children and adolescents. Am Fam Physician.

[2] Berkowitz MR (2013). Assessing impact of not attending lectures on osteopathic medical student performance: brief survey of the literature and proposed future research. Int J Osteopath Med.

[3] Wright NS, Gragg MN, Cramer KM (2011). Encouraging undergraduate class participation: a student perspective. CELT.

[4] Zhang B, Yu Y (2020). Analysis of the process control and teaching outcome of online classroom (in Chinese). Res Higher Edu Eng.

[5] Li X, Sun K, Cao K (2021). Remote online teaching practice of power engineering test technology course (in Chinese). Henan Agric..

[6] Ilková V, Ilka A (2016). Legal cybernetics: an educational perspective. IFAC-Papers Online.

[7] Cantrell T (1973). A cybernetic approach to university teaching. Br J Med Educ.

[8] Kinley D, Pradhan S (2022). Exploring the relationship between class participation and student performance in science. BJRD.

[9] Purnama A (2017). Incorporating memes and Instagram to enhance student’s participation. Lang Lang Teach.

[10] Zhang S (2002). Application of cybernetics to improve the teaching quality of physiology. J Higher Corresp Edu (Natural Sciences).

[11] Peña-Ayala A, Cárdenas-Robledo LA (2019). A cybernetic method to regulate learning through learning strategies: a proactive and reactive mechanism applied in U–learning settings. Comput Hum Behav.

[12] Terekhov AN, Luchin RM, Filippov SA (2012). Educational cybernetical construction set for schools and universities. IFAC Proc.

[13] Zhao LQ (2008). Brief talking about the influence of cybernetics on teaching process. J Teach Manage.

